# Management and Prevention of Traveler’s Diarrhea: A Cross-Sectional Study on Knowledge, Attitudes, and Practices in Italian Occupational Physicians (2019 and 2022)

**DOI:** 10.3390/tropicalmed7110370

**Published:** 2022-11-11

**Authors:** Matteo Riccò, Alessandro Zaniboni, Elia Satta, Antonio Baldassarre, Milena Pia Cerviere, Federico Marchesi, Simona Peruzzi

**Affiliations:** 1 Servizio di Prevenzione e Sicurezza Negli Ambienti di Lavoro (SPSAL), Department of Public Health, AUSL-IRCCS di Reggio Emilia, I-42122 Reggio Emilia, Italy; 2 Department of Medicine and Surgery, University of Parma, I-43126 Parma, Italy; 3 Department of Experimental and Clinical Medicine, University of Florence, I-50134 Florence, Italy; 4 Università Cattolica del Sacro Cuore, I-00168 Rome, Italy; 5 Laboratorio Analisi Chimico Cliniche e Microbiologiche, Ospedale Civile di Guastalla, AUSL-IRCCS di Reggio Emilia, I-42016 Guastalla, Italy

**Keywords:** surveys and questionnaires, health knowledge, attitudes, practice, physicians, primary care, referral and consultation, travel medicine, tropical medicine

## Abstract

Even though Italian Occupational Physicians (OP) are increasingly involved in the managing of overseas workers, their knowledge, attitudes, and practices (KAP) in travel medicine are mostly undefined. We, therefore, permed a KAP study specifically targeting the management of travelers’ diarrhea (TD) by OP. A total of 371 professionals (43.4% males; mean age 40.8 ± 10.9 years) completed in 2 rounds (2019 and 2022) a specifically designed web questionnaire that inquired participating OP on their knowledge status (KS), risk perception, and management of TD through pre- and post-travel advice and interventions. Multivariable odds ratios (aOR) for predictors of a better knowledge status were calculated through regression analysis. Eventually, the majority of participants (53.4%) had participated in the management of cases of TD in the previous months, but only 26.4% were reportedly involved in pre-travel consultations. The overall knowledge status was unsatisfying (potential range: 0–100%, actual average of the sample 59.6% ± 14.6), with substantial uncertainties in the management of antimicrobial treatment. Interestingly, only a small subset of participants had previously prescribed antimicrobial prophylaxis or treatment (3.5% and 1.9%, respectively). Main effectors of a better knowledge status were: having a background qualification in Hygiene and Public Health (aOR 14.769, 95%CI 5.582 to 39.073), having previously managed any case of (aOR 3.107, 95%CI 1.484 to 6.506), and having higher concern on TD, reported by acknowledging high frequency (aOR 8.579, 95%CI 3.383 to 21.756) and severity (aOR 3.386; 95%CI 1.741 to 6.585) of this disorder. As the adherence of participating OP to official recommendations for TD management was unsatisfying, continuous Education on Travel Medicine should be improved by sharing up-to-date official recommendations on appropriate treatment options for TD.

## 1. Introduction

Travelers’ diarrhea (TD) has been defined as the increase in the frequency of bowel movements during a trip abroad, up to three or more loose stools per day [1,2,3,4]. Considered the most predictable travel-related illness, it is a relatively common and self-limited condition, with attack rates ranging between 10% and 50% of all travelers visiting low-income countries, due to insufficient hygienic conditions, without neglecting the average warmer climate throughout the year [1,2,3,5,6], and usually lasting from 3 to 5 days without the need of specific etiologic treatments [2,5]. Traveler’s diarrhea is characterized by a syndromic corollary due to a great variety of intestinal pathogens, among which bacteria play a predominant role, being responsible for almost 80–90% of cases; viruses may account for at least 5–15% of events, while protozoal pathogens collectively account for approximately 10% of diagnoses [4,7]. Among bacteria, enterotoxigenic *Escherichia coli* (ETEC) has been historically recognized as the most common cause of TD, but in recent years its prevalence has declined worldwide, with increased occurrence of cases associated with enteroaggregative *E. coli* (EAEC) and enteropathogenic *E. coli* (EPEC) [4,7]. TD occurs equally in male and female travelers and is more common in young adult travelers than in older travelers [4].

During the last decade, global improvements in sanitation and hygiene infrastructures have led to a substantial decline in TD incidence rates [2,5,6,8,9,10]. However, these trends do not apply homogeneously to all travel populations, stressing how individual habits and travel environments may play a prominent role [6]. Following the inception of the SARS-CoV-2 pandemic, international tourist arrivals have dramatically dropped from 1.466 billion in 2019 to 409.5 million in 2020 and 446.2 million in 2021, but international agencies expect a substantial increase in the coming years, potentially targeting the goal of 1.8 billion by 2030 [11], underscoring the importance of sharing and applying evidence-based guidelines on prevention and treatment of TD [1,2,3,5].

In fact, while fatal cases have been only anecdotally reported [11], TD is associated with a substantial economic impact, including lost in time and productivity and unwanted medical expenses in terms of overseas medical emergencies and eventual hospitalizations [3,6,9,10]. Moreover, long-term sequelae are not uncommon, as up to 17% of cases may eventually develop a post-infectious irritable bowel syndrome (IBS) [2,3,4,5,12].

TD has acquired a distinctive significance in occupational settings, as individuals afflicted by TD during their travels are also precluded from participation in daily work-related activities [6,11,13,14]. Particularly where appropriate medical support may be quite difficult to obtain and rely on because of linguistic and/or infrastructural issues, or even remoteness, Occupational Physicians (OP) may represent key players in implementing an appropriate preventive and on-site management of TD in overseas workers [15,16,17]. Where implemented by legal frameworks, as for Italian Occupational Health and Safety Legislation, OP represent the medical professionals responsible for health promotion in the workplaces, participating in the workers’ formation [18,19], and sharing appropriate information about the pros and cons of recommended medical interventions [19,20]. Unfortunately, not only are guidelines on the prevention and treatment of TD quite heterogeneous and even contradictory [1,2,3,5,21], particularly when dealing with the use of antibiotics [5,21], but previous studies have also reported that OP may be affected by a significant misunderstanding on the managing of biological risk factors and infectious diseases [19,20,22,23,24,25], including TD [26].

In this regard, it should be stressed that our understanding of knowledge, attitudes, and practices (collectively KAP) of medical professionals on TD is mostly based on surveys from professionals working in academic or tertiary care units specialized in infectious/tropical diseases and travel medicine [12,27,28,29]. In other words, the actual familiarity with up-to-date and evidence-based preventive and clinical options of all other medical professionals, including OP but also General Practitioners (GP), still remains unclear [12,28,29,30].

In a preliminary study that we performed shortly before the inception of the SARS [26], we specifically focused on the KAP of a sample of Italian GPs, being able to stress how scarce was the current understanding of updated official recommendations on TD, particularly when dealing with the real-world risks associated with TD and unnecessary antibiotic treatments. Interestingly, this preliminary study included a small subset of GPs who also worked as OP, who not only contributed to the validation of the questionnaire (Table A2) but suggested that the basic knowledge gaps of Italian GPs on TD may be shared by OP. This follow-up study was therefore specifically designed in order to assess the KAP on TD in a larger sample of Italian OP, focusing on (a) their contribution to pre-travel advice and post-travel treatment; (b) how they use and recommend antimicrobial therapy; (c) their most relevant knowledge deficits.

## 2. Materials and Methods

### 2.1. Study Design and Population

This cross-sectional questionnaire-based study was originally designed as a follow-up study to our original survey on GPs in the Emilia Romagna region of Italy [26] (see STROBE (Strengthening the reporting of observational studies in epidemiology) checklist as Table A1 statement [31]). The questionnaire was originally delivered between 15 July 2019 and 15 August 2019 through a closed discussion group whose application was officially limited to OP. At the time, the group had approximately 2000 unique members, but no information could be obtained regarding the actual share of members actively participating in the group. Following the inception of the SARS-CoV-2 pandemic and its severe impact on the daily duties of OP [32,33], including the temporary suspension of work-related overseas travels [33,34], between 15 July and 15 August 2022, we performed a second round of delivery in five closed discussion groups whose admission was limited to licensed Italian medical professionals. In total, the groups had 10,293 unique members, but again no information could be obtained regarding how many of them were active participants, nor on how many of the participants were qualified as OP and/or were actively working as OP at the time of the survey.

In both rounds of delivery, the principal investigator (MR) initially contacted the administrators of the recipient group, requesting their preventive authorization for sharing the study invitation. The latter included:

(a) A summary of the aims of the survey;
(b) A link to the full informed consent, whose acceptance through a specific dichotomous question (yes vs. no) was mandatory for receiving;
(c) The direct link to the questionnaire (Google Forms, Google LLC, Menlo Park, CA, USA);
(d) A preliminary question about the respondent’s occupational settings, i.e., whether he/she was actually working as OP or not.

The questionnaire was made available to all group members receiving the questionnaire and agreeing with the participation during the time period mentioned above and once more between 15 July and 15 August 2022, but only participants who reportedly worked as OP at the time of the survey were able to complete it.

### 2.2. Questionnaire

The questionnaire was previously validated in a preliminary study performed on GPs [26], and it has been extensively described elsewhere. Briefly, it encompassed a total of 26 items divided into the following areas of inquiry:

#### 2.2.1. Characteristics of the Participants

Demographic characteristics of the participant were retrieved, including age, gender, seniority, and any previous personal experience with TD [35,36,37]. More precisely, participants were requested whether: (a) they had previously been affected by TD (ever vs. never), (b) they had been involved in any preventive consultation for TD in the previous 12 months (yes vs. no), (c) they had managed at least one case of TD in the last 12 months (yes vs. no). Participants were also asked whether they worked as General Practitioner alongside their activity as OP (yes vs. no). As the Italian legal framework guarantees to the specialists in Hygiene and Public Health (i.e., professionals having a presumed higher familiarity with management and prevention of infectious diseases) a conditioned qualification as OP, their status was also inquired through a dichotomous question (i.e., having or not a background qualification in Hygiene and Public Health).

#### 2.2.2. Knowledge Status

Participants were inquired about some common misconceptions about TD through a series of 20 true–false statements (e.g., “Usually, travelers’ diarrhea resolves spontaneously in 3 to 5 days”; TRUE) and 3 multiple-choice questions focusing on the etiology of watery diarrhea, dysentery, and gastrointestinal syndromes with diarrhea and vomiting (i.e., amoebic, fungal, Campylobacter spp., Cryptosporidium spp., *Escherichia coli*—ETEC, *Shigella* spp., *Vibrio cholerae*, Norwalk virus (NoV). All included items had been previously designed through accurate analysis of similar studies and Guidelines of the International Society of Travel Medicine available at the time of the pilot study (2019) [2,21,26]. A summary General Knowledge Score (GKS) resulted from the sum (+1 score) of all correctly marked statements, whereas a wrong indication or a missing/“don’t know” answer added 0 to the sum score. Similarly designed knowledge tests have previously been successfully applied in several KAP studies, particularly when dealing with biological risk factors [18,19,20,25,38,39,40]. GKS (potential range of 0–23) was then normalized in a percent value and dichotomized by the median in “high knowledge status” (>median value) vs. “low knowledge status” (≤median value).

#### 2.2.3. Risk Perception

Perceived risk represents a significant effector for eventual behaviors [18,35,36,37,41,42] and may be reported as a function of the perceived probability of a certain event and the severity of its expected consequences [18,20,25,39,43]. Participants were therefore asked to rate how they perceived the incidence (INF) and the potential severity (SEV) of TD in occupational settings through a 5-point Likert scale (i.e., 1 = low concern, 5 = high concern). INF and SEV were initially dichotomized in acknowledging some concerns (i.e., score 4–5) vs. not concerned (score 1–3). A cumulative Risk Perception Score (RPS) was then calculated as the mathematical product of the perceived incidence and severity of TD (i.e., RPS = INF × SEV).

#### 2.2.4. Practices

We specifically inquired OP participating in our study whether, in the previous 12 months, they had recommended any antimicrobial prophylaxis to travelers and antimicrobial treatment in suspected cases of TD. Both items were assessed by means of a fully labeled 5-point Likert scale that ranged from “never” (i.e., the participants deliberately excluded the use of antimicrobial prophylaxis/treatment in all consultations), to “rarely” (i.e., antimicrobial prophylaxis/treatment is usually avoided, but rare exceptions are possible), “sometimes” (i.e., prophylaxis/treatment is usually avoided, but selected cases may still be targeted), “often” (i.e., prophylaxis/treatment is recommended in the majority of travelers, according to their clinical conditions and environmental features of the travel), and “always” (i.e., prophylaxis/treatment is recommended to all travelers).

### 2.3. Data Analysis

A preliminary check on collected data was performed by two researchers who independently ensured their consistency, while the primary investigator examined unclear responses to arbitrarily determine the correct answer. Partially completed questionnaires or questionnaires lacking demographic data were excluded from eventual analyses.

In the descriptive analysis, continuous variables were initially expressed as mean ± Standard Deviation (SD) and tested for normal distribution by means of the D’Agostino and Pearson omnibus normality test (cut-off value *p* < 0.100 for rejecting normal distribution), while categorical variables were reported as percent values. Normally distributed continuous variables were compared by means of Student’s *t*-test or ANOVA, where appropriate, while not normally distributed continuous variables were compared through Mann–Whitney or Kruskal–Wallis tests for multiple independent samples. Similarly, the association between continuous variables was assessed through Pearson’s correlation coefficient (normally distributed variables) or Spearman’s rank correlation coefficient (not normally distributed variables).

Before any further analysis, the internal consistency of the questionnaire (i.e., how closely related a set of items are as a group) was assessed by calculation of Cronbach’s alpha coefficient of reliability on the items of the knowledge test. Values of Cronbach’s alpha range from 0 to 1, and although the standard for considering the estimate as “good” or conversely “unacceptable” are entirely arbitrary, many methodologists recommend a minimum value ranging between 0.65 and 0.8 (or higher in many cases) [44].

Association of individual factors (i.e., demographic factors, previous interactions with TD), personal attitudes (i.e., INF and SEV), individual practices (i.e., having or not recommended antimicrobial prophylaxis and/or treatment) with the higher knowledge status was initially evaluated through the Chi2 test. A multivariable regression analysis was then modeled with all variables that in univariate analysis were associated with higher knowledge status with a *p* value < 0.05 and corrected for the following factors: gender, age, having previously managed any RD case and having participated in the survey during 2022 vs. 2021. Adjusted Odds Ratios (aORs) with their respective 95% confidence intervals (95% CI) were calculated accordingly (SPSS 26, IBM Corp., Armonk, NY, USA).

### 2.4. Ethical Considerations

The study was conducted according to the guidelines of the Declaration of Helsinki. A preventive ethical review and approval were waived for this study because of its anonymous, observational design and due to the lack of clinical data about patients that could configure the present research as a clinical trial. Participants were also guaranteed that retrieved data would be stored only for the time required by data analysis. The study, therefore, did not configure itself as a clinical trial, and a preliminary evaluation by an Ethical Committee was not required, according to Italian law (Gazzetta Ufficiale no. 76, dated 31 March 2008).

## 3. Results

### 3.1. Individual Characteristics of Respondents

**Figure 1:**
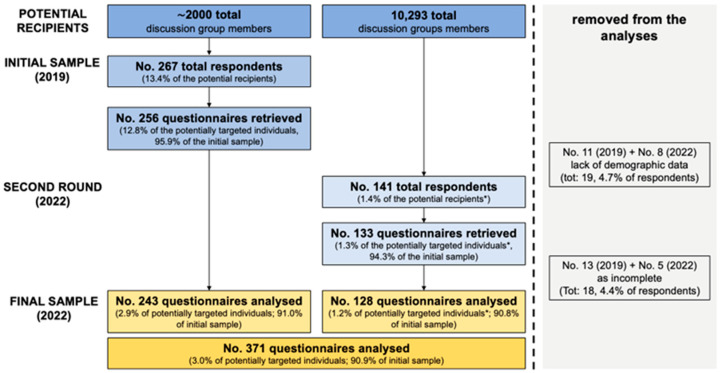
Flow chart of the study participants, Italy 2019 and 2022. Note: * = total number of Occupational Physicians included in the parent groups not available; the share of potentially targeted individuals is therefore calculated on the total number of group members.

As shown in Figure 1, in 2019, a total of 243 participants were recruited (i.e., 2.9% of potentially targeted OP from the parent discussion group and 91.0% of the initial sample of 267 retrieved questionnaires). On the contrary, in 2022, a total of 141 respondents participated in the survey (1.4% of potential recipients), and 128 questionnaires were ultimately included in the analyses (1.2% of potentially targeted individuals and 90.8% of the initial sample).

In this regard, it should be stressed that while the parent group 2019 only included OP, the discussion groups targeted in the second round of 2022 was open to all medical professionals, and the actual number of the active OP was unknown at the time of the survey.

In summary, a total of 371 participants were included in the analyses (Table 1). The mean age was 40.8 years ± 10.9, with no substantial difference between 2019 and 2022. The majority of them were of the female gender (56.6%), and the corresponding share increased from 53.5% in 2019 to 62.5% in 2022 (*p* = 0.096). The average seniority as OP of respondents was 13.3 years ± 11.7, and again no substantial differences were identified between respondents in 2019 vs. 2022 (13.2 years ± 12.5 vs. 13.6 years ± 10.1, *p* = 0.100). However, the share of participants reporting a professional experience as OP ≥ 10 years at the time of the survey increased from 69.1% in 2019 to 75.8% in 2022 (*p* = 0.002).

Of them, 13.5% reportedly worked as General Practitioners alongside their main activity as OP, with no differences between 2019 and 2022, while the share of professionals having a background in Hygiene and Public Health (10.5% for the sample as a whole) was greater among participants from 2019 (14.0%) than in those from 2022 (3.9%, *p* = 0.003).

Focusing on the previous professional interaction with TD, around 1/3 of participants were previously affected by this disorder (36.1%), with no substantial differences among participants recruited in 2019 (35.0%) compared to 2022 (38.3%, *p* = 0.529). On the contrary, the share of respondents having managed at least one case of TD in the previous 12 months decreased from 56.4% in 2019 to 47.7% in 2022 (*p* = 0.035), for a pooled estimate of 53.4%. Similarly, participants having been involved in preventive consultations in the previous 12 months decreased from 32.1% in 2019 to 15.6% in 2022 (*p* < 0.001, pooled estimate: 26.4%).

### 3.2. Knowledge Status

**Figure 2:**
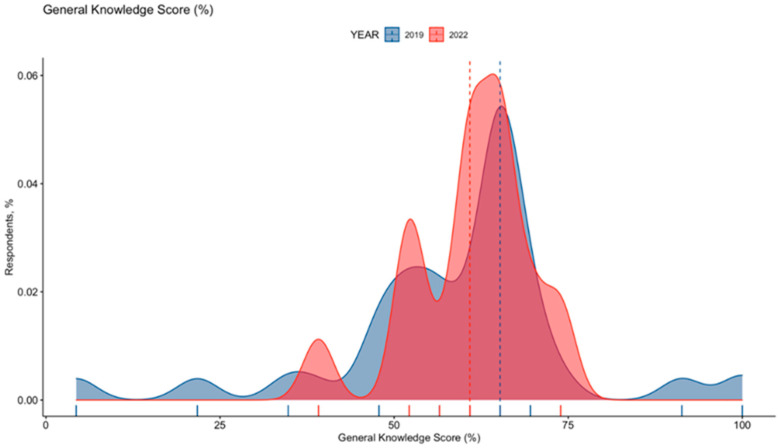
General Knowledge Score (GKS) on Travelers’ Diarrhea in 371 Italian occupational physicians participating in the survey, broken down by year of recruitment (i.e., 2019 vs. 2022). GKS was substantially skewed (D’Agostino–Pearson’s normality test K2 = 151.6, *p*-value < 0.001). Dotted lines represent median value.

As shown in Table 1 and Figure 2, the overall estimate for GKS of 59.6% ± 14.6 (actual range 4.4% to 95.7%) was characterized by a not significant increase from 58.9% ± 16.9 in 2019 to 61.0% ± 8.5 in 2022 (*p* = 0.334), being substantially skewed and not normally distributed (D’Agostino–Pearson’s normality test K2 = 151.6, *p*-value < 0.001).

The determination of Cronbach’s alpha resulted in an estimate of 0.707, suggesting an acceptable internal consistency of the questionnaire. Single statements are reported in Table 2, for all the respondents and for those completing the questionnaire in 2022 compared to 2019. Briefly, the large majority of respondents acknowledged TD as a benign disorder (Q1) that resolves spontaneously in 3–5 days (84.4%) and reported an appropriate understanding of some basic preventive interventions, including the use of high temperature on fluids and beverages (Q9; 94.3%), and the residual risk associated with fresh fruits and/or vegetables (Q18; 92.7%), ice drinks (Q10; 94.3%), and drinks on taps (Q19; 89.5%). Interestingly, a better understanding of most of such items was reported in 2022 compared to 2019 (Q1 and Q9: 93.8% vs. 79.4%, *p* < 0001; Q10 100% vs. 91.4%, *p* = 0.001; Q18 100% vs. 88.9%, *p* < 0.001). Some significant uncertainties were reported on the epidemiological features of TD, as only 27.2% had knowledge that in the last decades, the global incidence of TD is actually decreasing (Q21, 27.2%, with 35.9% of correct answers in 2022 compared to 22.6% in 2019, *p* = 0.006), but up to two-thirds of participants correctly identified in Southern Asia as the area at highest risk for TD (Q22, 67.1%), and the wilderness travelers as individuals at particularly high risk for developing this disorder (Q11, 62.0%). Interestingly, the awareness of the high risk for TD in Southern Asia decreased from 74.5% in 2019 to 53.1% in 2022 (*p* < 0.001).

As regards treatment options, most respondents correctly acknowledged fluoroquinolones as antibiotics that can be used as first-line options for TD (Q4; 79.0%), while only one third of participants understood that these antibiotics are not specifically recommended for patients at high risk of complications (Q2; 36.7%). Moreover, around 60% of participating OP correctly identified rifaximin as a preventive option for patients at high risk for complications (Q3, 58.8%) and as a therapeutic option for severe, non-dysenteric TD (Q5, 62.3%). Alternative non-antimicrobial treatment was similarly associated with a mixed pattern of understanding, as 59.0% acknowledged the lack of consolidated evidence for recommending the use of probiotics as a preventive option (Q6, 59.0%; increasing from 53.9% in 2019 to 68.8% in 2022, *p* = 0.006), and 62.3% correctly recalled the proper use of loperamide and analogous in managing of TD (Q16, 62.3%; 53.5% in 2019 compared to 78.9% in 2022, *p* < 0.001), but only 29.1% had any familiarity with the use of bismuth subsalicylate (Q17, 41.6% in 2019 compared to 5.5% in 2022, *p* < 0.001).

When dealing with available vaccination strategies, many participants acknowledged the availability in Italy of a typhoid vaccine (Q20, 90.6%), while substantial uncertainties were reported on the reliability of anti-cholera (Q7; 45.6% of correct answers, increasing from 37.4% in 2019 to 60.9% in 2022; *p* < 0.001), and particularly anti-rotavirus immunizations (Q8; 35.0%) immunizations for preventing TD. In fact, knowledge gaps appeared concerning TD etiology. Even though 59.8% of the sample correctly identified the etiologic role of bacterial infections (Q12, increasing from 55.1% in 2019 to 68.8% in 2022), only 35.6% identified in *Shigella* spp. the most frequently reported cause of dysentery (Q14; decreasing from 45.7% in 2019 to 16.4% in 2022, *p* < 0.001), while a larger share of participating OP improperly recalled this role for ETEC (36.9%), followed by *Campylobacter* spp. (10.8%), *Vibrio cholerae* (7.3%), NoV (5.7%), and amoebic infections (3.8%). Similarly, only 40.7% of OP correctly identified ETEC as the main cause of watery diarrhea (Q13), followed by *Campylobacter* spp. (19.9%), NoV (12.9%), *Vibrio cholerae* (11.3%), and eventually *Shigella* spp. (9.7%), and amebic infections (5.4%). Finally, only a quarter of participants (i.e., 25.9%) correctly associated NoV with a syndrome characterized by diarrhea and vomiting (Q15), whereas the majority of respondents identified the main etiology in other pathogens, such as *Shigella* and *Campylobacter* spp (for both pathogens, 18.9%), followed by ETEC (12.9%), *Vibrio cholerae* (11.9%), amoebic infections (8.6%), and *Cryptosporidium* spp (1.6%).

### 3.3. Risk Perception

Overall, 60.1% of participants perceived TD as a condition that in overseas workers can be acknowledged as frequent or highly frequent, with no substantial differences between 2019 (60.5%) and 2022 (59.4%, *p* = 0.834). On the contrary, only one-third of all respondents acknowledged TD as a severe or even highly severe condition (32.9%), and even though the corresponding share increased in 2022 compared to 2019 (36.0% vs. 31.3%), the difference was not substantial (*p* = 0.364).

**Figure 3:**
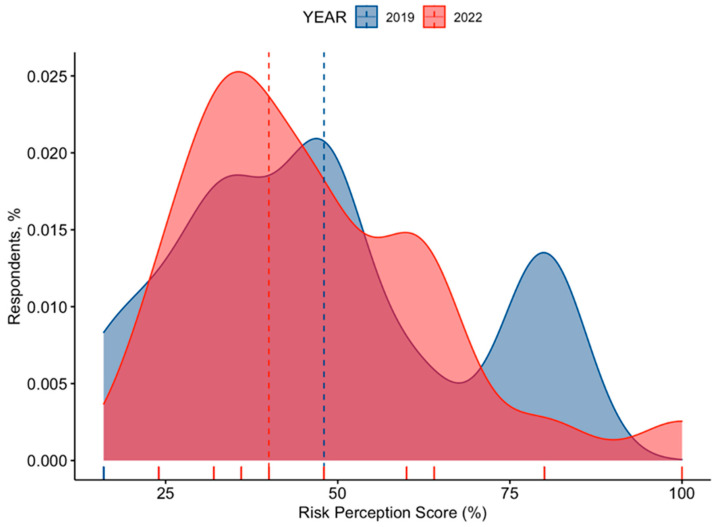
Risk Perception Score (RPS) on Travelers’ Diarrhea in 371 Italian occupational physicians participating in the survey, broken down by year of recruitment (i.e., 2019 vs. 2022). RPS was substantially skewed (D’Agostino–Pearson’s normality test K2 = 21.9, *p*-value < 0.001). Dotted lines represent median value (actual range: 16 to 100).

Corresponding RPS was estimated to be 47.1% ± 19.5 (actual range: from 16.0% to 100%). As shown in Figure 3, the score was substantially skewed (D’Agostino–Pearson’s normality test K2 = 21.9, *p*-value < 0.001), with no substantial differences between estimates for 2022 (46.5% ± 18.0) and 2019 (47.4% ± 20.4, *p* = 0.724).

### 3.4. Univariate Analysis

**Figure 4:**
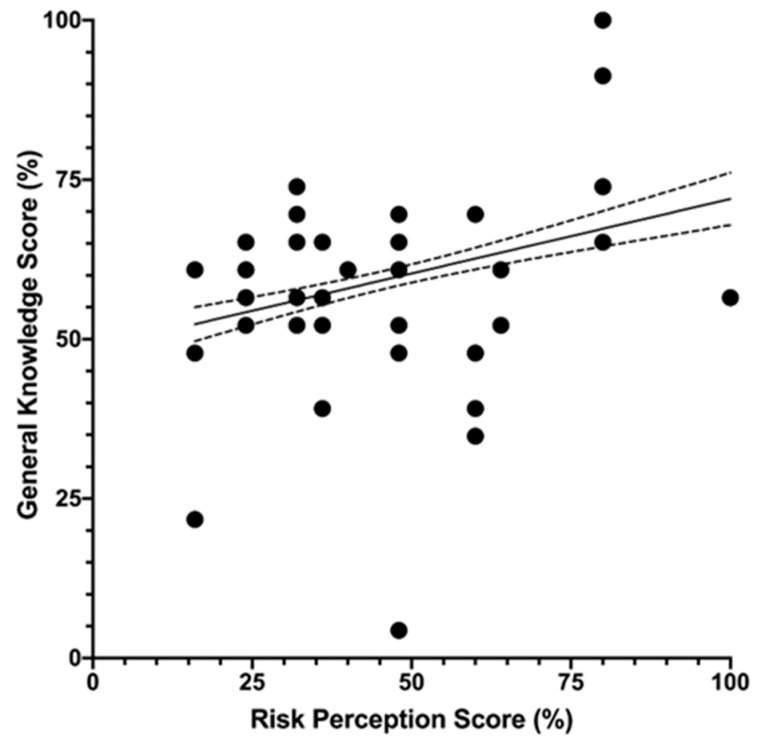
Correlation between General Knowledge Score (GKS) and Risk Perception Score (RPS) on Travelers’ Diarrhea in 371 Italian occupational physicians participating in the present survey (2019, 2020). GKS and RPS were ultimately well correlated (Spearman’s rho = 0.286, *p* < 0.001).

As shown in Figure 4, RPS and GKS were positively well correlated (Spearman’s rho = 0.286, *p* < 0.001): in other words, individuals characterized by a better understanding of TD reported a higher risk perception of this disorder.

In univariate analysis (Table 3), a better knowledge status was positively associated with the male gender (62.7% vs. 39.1% among respondents with low knowledge status, *p* < 0.001), reporting a background qualification in Hygiene and Public Health (26.9% vs. 6.9%, *p* < 0.001), recognizing TD as a frequently reported (89.6% vs. 53.6%, *p* < 0.001), and severe disorder (49.3% vs. 29.3%, *p* = 0.002) among overseas workers.

### 3.5. Multivariable Analysis

In multivariable analysis, the outcome variable of better knowledge status on TD was assessed through a binary logistic regression model that included the following explanatory variables (Table 4): male gender, age, having previously managed TD, having a background qualification in Hygiene and Public Health, recognizing TD as a frequently reported disorder, recognizing TD as a severe disorder, and having fulfilled the questionnaire in 2022.

Eventually, the higher knowledge status was positively associated with reporting a background qualification in Hygiene and Public Health (aOR 14.769, 95%CI from 5.582 to 39.073), having managed any case of TD in the previous 12 months (aOR 3.107, 95%CI from 1.484 to 6.506), recognizing TD as a frequently reported (aOR 8.579, 95%CI from 3.383 to 21.756) and severe disorder (aOR 3.386; 95%CI from 1.741 to 6.585).

## 4. Discussion

In our cross-sectional study on 371 Italian OP, the actual knowledge and risk perception of sampled participants on TD were far from optimal, suggesting that substantial formative interventions on travel medicine should be implemented in order to improve the capability of these professionals to properly cope with the requirements of overseas workers. As suggested by previous studies on frontline healthcare professionals, knowledge status is critical for guaranteeing the appropriate spreading of preventive measures and updated management options for TD [3,12,26,30,45,46,47,48,49], and also in our study, knowledge status and risk perception were well correlated, stressing how an improved understanding of TD may lead to a better perception of potential health issues associated with this disorder, potentially improving the health status of workers referring to OP for pre- and post-travel advice. More precisely, effective predictors of higher knowledge status were identified in a background qualification in Hygiene and Public Health (aOR 14.769, 95%CI 5.582 to 39.073), having previously managed any TD case (aOR 3.107, 95%CI 1.484 to 6.506), ad having high concern on TD, reported by acknowledging its frequency (aOR 8.579, 95%CI 3.383 to 21.756) and severity (aOR 3.386; 95%CI 1.741 to 6.585).

While the better understanding of TD in professionals having a background qualification in Hygiene and Public Health can be explained through the greater familiarity of those professionals with infectious diseases [19,25,40,50,51], all the remaining factors and their role in modeling a better understanding of TD issues may find a reasonable and straightforward explanation in the Health Belief Model (HBM) [52,53]. HBM was developed in the 1950s and remains quite effective in explaining and predicting individual changes in health behaviors [35,36,37] through the basic assumption that the beliefs about the susceptibility to a certain health threat, correspondent perceptions on the potential severity of that threat, and perceived benefits (and, conversely, barriers) associated with a particular intervention, will determine whether or not an individual would adopt that action [41,54]. As a consequence, having previously faced TD (either as a personal condition, or in friends, relatives, or even among a substantial share of patients) has reasonably molded an increased consideration of all the aspects associated with this disorder and, as shown in Appendix B (Table A3), individuals having any previous experience on TD were actually characterized by higher risk perception and knowledge status than those having not.

According to the current legal framework [50,55,56,57], Italian OP are actively involved in applying and tailoring preventive interventions in the workplaces [51,56]. When dealing with overseas workers, an appropriate pre-travel consultation represents a major opportunity to educate the worker about health risks at the destination and how to mitigate them [58], eventually improving travelers’ health status [59,60], representing and increasingly important field of intervention not only for GP [26,59,61] but also for OP [58,62]. Although the substantial health burden represented by TD in international travelers [1,2,47,63,64], including occupational settings and overseas workers [45], very little is known about the KAP of OP. Interestingly, even the share of OP actively involved in pre-travel consultation is largely undefined. In this study, around 1/4 of all respondents were actively involved in pre-travel consultation of overseas workers in the year before the collection of the survey, and this share has substantially decreased in 2022 compared to 2021 (15.6% vs. 32.1%). These figures can be easily explained as a consequence of the SARS-CoV-2 pandemic, including the implementation of an extensive travel ban that clearly affected work-related travel [34,65], but stress the improperly low implementation of this potentially useful intervention. In fact, our study specifically targeted TD, a mostly benign condition, but through pre-travel consultations, more insidious issues can be properly targeted, including the enactment of mandatory vaccinations (ranging from yellow fever to the requirements for meningococcus and SARS-CoV-2 immunizations) [13,66,67], the tailoring of malaria prophylaxis according to international guidelines and baseline clinical conditions of the traveler where needed [13,67], and providing basic recommendations for travels in areas characterized by increased health risks.

Unsurprisingly, TD represented a considerable issue not only for professionals actively involved in pre-travel consultations: even though the share of respondents who had managed at least one case of TD in the previous 12 months was greater among professionals participating in pre-travel consultation than in those who were reportedly not involved (79.6% vs. 44.0%), the resulting amount of time spent in these activities was substantial (44.0%; Table A4). On the contrary, the high share of professionals dealing with TD in the year before the collection of the questionnaire (i.e., 53.4%) was quite unexpected. Italian OP are usually considered professionals only focusing on preventive interventions, with very a very limited clinical role [20,39,50,51], but our study suggests that OP may be involved in some clinical issues in terms quite similar to GPs and other frontline providers [12,26,49,68]. From this point of view, it is important to stress that the knowledge status of recruited professionals was largely unsatisfying, with a significant proportion of uncertainties and false beliefs on various aspects of TD. For instance, overall understanding of TD-related issues was unsatisfying, ranging from epidemiological and microbiological characteristics of such disorder to current recommendations for preventive options and antimicrobial treatment. Not only a very large share of participating OP had limited knowledge of the declining global incidence of TD, but substantial knowledge gaps affected the currently understanding of areas associated with higher risk for TD and the main causative microbial agents [12,29]. In this regard, the decreasing global occurrence of ETEC cases, with the progressive emergence as lead pathogens of EAEC and EPEC, has possibly led some of the respondents to improperly deny ETEC as a leading cause of TD [7,9]. Future iterations of this study should, therefore, more specially address the understanding of ETEC/EAEC/EAEC in the global epidemiology of TD, according to up-to-date figures. Similarly, as recently stressed by Butler et al. [4], the risk of developing TD varies within countries, particularly for larger countries such as Brazil and China, and this gradient is not only scarcely discussed in the literature but may have been improperly perceived by participants [4].

In fact, available options for primary prevention through effective vaccines were affected by extensive misunderstandings. Even though reasonably effective vaccines have been made available against *S. typhi*, *V. cholerae,* and rotavirus, none of these agents is acknowledged as a major cause of TD [5], and *S. typhi* vaccination is only recommended for travelers to endemic settings [2,3,5,21].

Moreover, only a very limited subset of recruited OP actually recommended antimicrobial treatment either as a preventive or treatment option [3,9,10,12,29,48], and their understanding of official recommendations appeared particularly outdated. Italian Guidelines on TD were issued more than a decade before the first round of this study (i.e., 2005) [26], but a series of updated international guidelines [2,3,4,9] have been issued. The cornerstone of TD guidelines is recommending a cautious but targeted use of certain antibiotics (e.g., rifaximin) in order to ease the prognosis and to reduce the consequences of acute diarrhea, particularly in high-risk groups [2,3,5], reducing the potential spread of antimicrobial resistances [2,12,48] and avoiding colonization of travelers by resistant organisms [28].

Nonetheless, participants were improperly reluctant to rely on antimicrobial treatment (i.e., 3.5% recommended any preventive treatment, and only 1.9% recommended any antimicrobial treatment), with figures that were hardly comparable to our previous study on Italian GPs [26], and also to similar studies on the frontline and primary care providers [28,30], as nearly all medical professionals involved in pre-travel counseling usually recommend to preventively buy antibiotics among suitable medications [68,69]. Again, some explanation may be found in the institutional status of Italian OP: as mostly free practitioners [51,57], Italian OP usually face substantial constraints in managing treatment options that are strictly monitored from the central level, including the prescription of antibiotics [70,71]. However, the knowledge test identified substantial uncertainties in the use of specific medications. This is of particular concern as only in recent years has travel medicine been implemented in the core curriculum of university levels and some post-graduation (i.e., specialization) courses, including those focusing on Occupational Medicine [26,50,51,56], while it historically represents a cornerstone of the qualification in Hygiene and Public Health, that was associated with better performances in knowledge test and was also identified as a substantial effector for a better knowledge status [51,56]. In other words, until recently, being up-to-date on topics specifically associated with travel medicine, including an appropriate understanding of preventive and managing options for TD, has substantially depended on the individual and voluntary participation of a specific medical professional in Continuous Medical Education activities, with a residual high risk for significant knowledge gaps. Not coincidentally, the knowledge test hints towards a highly shared confidence in a “wait-and-see” approach to TD, with high confidence in symptomatic interventions with loperamide that was mirrored by the ignorance of evidence-based recommendations for non-antimicrobial medications, including bismuth subsalicylate [2,3,5,21], and probiotics [72], while antibiotic treatment was seemingly reserved only to, particularly severe TD or diarrhea occurring in patients at high risk of systemic complication [2,3,5,21,68,69].

The knowledge gaps on bismuth subsalicylate and probiotics may be particularly informative about the shared unfamiliarity with updated recommendations on the management of TD among the recipients of our survey. Some evidence suggests that bismuth subsalicylate may be an appropriate option in the management of mild symptoms of TD, eventually delaying or even avoiding the requirements for antibiotic treatments [2,3,5,21]. Still, not only were most of the participants unaware of the potential benefits from this treatment (around 70%), but also the share of OP having any knowledge of the rationale behind bismuth subsalicylate decreased from 2019 to 2022, potentially suggesting the widening of this knowledge gap. Conversely, around 40% of participants appeared somewhat overconfident regarding the use of probiotics in TD prevention and treatment. Even though the corresponding share decreased, some meta-analyses have shown that probiotics from 2019 to 2022, these figures remain quite unsatisfying as there is substantial evidence that the effect of probiotics on TD may be marginal [72,73,74].

*Limits*. Despite the potential significance of this study, several substantial shortcomings and limits must be addressed and discussed.

First of all, the present study has been designed as an Internet-based survey, whose shortcomings are therefore shared by our report [75,76]. Even though web surveys are increasingly appreciated because of their reliability, cost-effectiveness, and for being not time-consuming interventions, participants are often and extensively “self-selected”, with a final sample that over-represents certain sub-groups of the original population (i.e., subjects from younger age groups, with greater literacy, and who are more accustomed to internet access), with a significant selection bias, particularly when compared to more conventional surveys [20,25,39,43,77]. Likewise, participating in the survey could be due to a proactive attitude associated with a pre-existing interest in the addressed topic, with a baseline knowledge that may lead to the substantial overestimation of the actual knowledge status [18,40,78].

Second, even though we implemented a very flexible and reliable design for the knowledge test and risk perception assessment [18,20,38,39,43], we cannot rule out the possibility that the knowledge score may have been affected by a significant social desirability bias. In other words, participants may have rather reported the answers that they understood as “socially appropriated” than their authentic ones [79].

Third, not only was our study designed as a cross-sectional study, but our sample was also of limited size and should be only cautiously interpreted as representative of the Italian National level [21,31,32]. In fact, 371 OP represent around 4.8% of all officially registered Italian professionals (in total, 7722 by the end of January 2022), an estimate that can hardly be considered fully representative of the national level. In other words, the present study was hardly generalizable, particularly in a country, such as Italy, characterized by distinctive regional patterns, also considering school-distinctive training during the residency program in occupational medicine [51]. Moreover, while the Italian workforce is growing older, the mean age of the respondents ranged around 40 years at the time of the survey. As these figures are a decade lower than national estimates (i.e., around 55 years of age) [80], that collectively hints towards a substantial oversampling of younger age groups [29,30,32].

Fourth, it is important to stress that the collected data were not externally validated. Consequently, there were no reliable estimates on the TD cases that were actually treated by participating OP. More precisely, how the recommendations on the managing of TD have been actually shared with the affected workers and then implemented by potential recipients and shared with other healthcare providers still remains unclear.

Last but not least, the inception of the SARS-CoV-2 pandemic has led to the dichotomization of our study in two distinctive “rounds”. In order to avoid duplicated questionnaires, in 2022, we opted for sharing the questionnaire in discussion groups that were well distinctive from the original one. However, this choice has led to the potential inconsistency of the study populations, as otherwise suggested by some differences in the composition of the sample, particularly the share of professionals having a background in Hygiene and Public Health, and by the heterogeneous referral of several items at knowledge test. Such issues can be explained as an indirect consequence of the pandemic, whose requirement for professionals having a background in Hygiene and Public Health has reasonably led some professionals to shift from private practice in Occupational Medicine to other roles in Public Health settings [25,81,82]. Similarly, also the differences reported between 2019 and 2022 on the management of TD and pre-travel advice may be explained as a consequence of the travel ban during 2020 and early stages of 2021, being therefore independent of pre-existing differences between the targeted populations.

## 5. Conclusions

In conclusion, our data collectively hint towards active involvement of sampled Italian OP in pre-travel advice of overseas workers. Interestingly, the tasks they claimed seemingly exceeded the usual interventions of OP: even though Occupational Medicine is mostly acknowledged as a diagnostic and preventive branch of medicine, as well as recalled by the ICOH Code of Ethics (third edition 2014), they not only participated in pre-travel advice but also guaranteed post-travel management. As most of them are actively involved in the management of TD, improving the core competencies of OP on travel medicine represents an urgent need in a globalized world that is rapidly recovering from the constraints of the SARS-CoV-2 pandemic. This appears particularly needed, as substantial knowledge gaps were identified in the management of anti-microbial therapy. Again, the unbalance between the core curriculum of specialization courses in Occupational Medicine and their actual tasks may both explain such gaps and suggests that focused informative interventions on appropriate etiological (i.e., microbiological) prophylaxis and treatment of infectious diseases may be of some interest for the management of overseas workers. In times that require talking about global health, our study underlines the importance of the OP, increasingly called to play a crucial role in the protection of health and safety of workers, notably including overseas workers, and call for implementing specifical training programs on travel medicine and focused anti-microbial prophylaxis and therapy, both in graduate schools of Occupational Medicine and through Continuous Medical Education interventions.

## Figures and Tables

**Table 1:** General characteristics of the 371 Italian Occupational physicians in Italy participating in the study on travelers’ diarrhea (TD) (2019 vs. 2022). Continuous variables were compared by means of Mann–Whitney U test, while categorical ones were compared through chi-squared test.

	Total Sample (No. 371)	2019 (No. 243)	2022 (No. 128)	*p* Value
Age (years) (Average ± SD)	40.8 ± 10.9	40.8 ± 11.8	40.8 ± 9.1	0.469
				0.223
<35 years	106, 28.6%	75, 30.9%	31, 24.2%	
35–49 years	215, 58.0%	133, 54.7%	82, 64.1%	
≥50 years	50, 13.4%	35, 14.4%	15, 11.7%	
Gender				0.096
Male	161, 43.4%	113, 46.5%	48, 37.5%	
Female	210, 56.6%	130, 53.5%	80, 62.5%	
Seniority (years) (Average ± SD)	13.3 ± 11.7	13.2 ± 12.5	13.6 ± 10.1	0.100
				0.002
0–9 years	165, 44.5%	124, 51.0%	41, 32.0%	
10–19 years	141, 38.0%	84, 34.6%	57, 44.5%	
≥20 years	65, 17.5%	35, 14.4%	30, 23.4%	
Working as General Practitioner	50, 13.5%	36, 14.6%	14, 10.9%	0.299
Background qualification in Hygiene and Public Health	39, 10.5%	34, 14.0%	5, 3.9%	0.003
Professional interaction with TD				
Managed at least one case of TD in the last 12 months	198, 53.4%	137, 56.4%	61, 47.7%	0.035
Involved in preventive consultations in the previous 12 months	98, 26.4%	78, 32.1%	20, 15.6%	<0.001
Previously affected by TD	134, 36.1%	85, 35.0%	49, 38.3%	0.529
General Knowledge Score (%); (Average ± SD)	59.6 ± 14.6	58.9 ± 16.9	61.0 ± 8.5	0.334
Recognizing TD as a frequent/highly frequent disease	223, 60.1%	147, 60.5%	76, 59.4%	0.834
Recognizing TD as a severe/highly severe disease	122, 32.9%	76, 31.3%	46, 36.0%	0.364
Risk Perception Score (%); (Average ± SD)	47.1 ± 19.5	47.4 ± 20.4	46.5 ± 18.0	0.724
Practices (often to always)				
Recommends antimicrobial prophylaxis	13, 3.5%	13, 5.3%	0, -	0.008
Recommends antimicrobial treatment	7, 1.9%	7, 2.9%	0, -	0.053

**Table 2:** Items included in the knowledge test and corresponding correct answers from 371 physicians from northwestern Italy (internal consistency assessed through determination of Cronbach’s Alpha, value 0.707). Comparisons between 2019 and 2022 were performed by means of chi-squared test.

	Correct Answer	Total (No./371, %)	2019 (No./243, %)	2022 (No./128, %)	*p* Value
1. Usually, travelers’ diarrhea resolves spontaneously in 3–5 days	TRUE	313, 84.4%	193, 79.4%	120, 93.8%	<0.001
2. Fluoroquinolones are specifically recommended for patients at high risk of medical complications	FALSE	136, 36.7%	92, 37.9%	44, 34.4%	0.508
3. Rifaximin should be preventively employed in patients at high risk of complications	TRUE	218, 58.8%	139, 57.2%	79, 61.7%	0.401
4. Fluoroquinolone antibiotics should be avoided as first-line option in patients affected by travelers’ diarrhea	FALSE	293, 79.0%	186, 76.5%	107, 83.6%	0.113
5. Rifaximin may be used to treat severe, non-dysenteric travelers’ diarrhea	TRUE	231, 62.3%	152, 62.6%	79, 61.7%	0.875
6. There is insufficient evidence to recommend the use of commercially available prebiotics or probiotics to prevent or treat travelers’ diarrhea	TRUE	219, 59.0%	131, 53.9%	88, 68.8%	0.006
7. Anti-cholera immunization is somewhat protective for travelers’ diarrhea	TRUE	169, 45.6%	91, 37.4%	78, 60.9%	<0.001
8. Anti-rotavirus immunization is somewhat protective for travelers’ diarrhea	FALSE	130, 35.0%	91, 37.4%	39, 30.5%	0.180
9. Treating liquids/beverages at 100°C for 1′ reduces risk of travelers’ diarrhea	TRUE	313, 84.4%	193, 79.4%	120, 93.8%	<0.001
10. Consumption of ice drinks reduces risk of travelers’ diarrhea	FALSE	350, 94.3%	222, 91.4%	128, 100%	0.001
11. Travelers’ diarrhea affects up to 60% of wilderness travelers	TRUE	230, 62.0%	154, 63.4%	76, 59.4%	0.451
12. Most infections associated with travelers’ diarrhea are of bacterial etiology	TRUE	222, 59.8%	134, 55.1%	88, 68.8%	0.011
13. The most frequently reported cause of watery diarrhea is…	*Escherichia coli*	151, 40.7%	98, 40.3%	53, 41.4%	0.841
14. The most frequently reported cause of dysentery is…	*Shigella* spp.	132, 35.6%	111, 45.7%	21, 16.4%	<0.001
15. The most frequently reported cause of gastrointestinal syndrome characterized by diarrhea and vomiting is…	Norwalk virus	96, 25.9%	64, 25.9%	33, 25.8%	0.976
16. Use of loperamide and analogous is not supported by available evidence	FALSE	321, 62.3%	130, 53.5%	101, 78.9%	<0.001
17. Bismuth Subsalicylate (BSS) may be considered for any traveler to prevent travelers’ diarrhea	TRUE	108, 29.1%	101, 41.6%	7, 5.5%	<0.001
18. Fresh fruits/vegetables reduce risk of travelers’ diarrhea	FALSE	344, 92.7%	216, 88.9%	128, 100%	<0.001
19. Drinks on tap are associated with reduced risk of travelers’ diarrhea	FALSE	332, 89.5%	216, 88.9%	116, 90.6%	0.604
20. A typhoid vaccine is available in Italy	TRUE	336, 90.6%	215, 88.5%	121, 94.5%	0.058
21. Globally, incidence of travelers’ diarrhea is decreasing	TRUE	101, 27.2%	55, 22.6%	46, 35.9%	0.006
22. The geographic area at highest risk of travelers’ diarrhea is Southern Asia	TRUE	249, 67.1%	181, 74.5%	68, 53.1%	<0.001
23. Risk of travelers’ diarrhea is usually higher in Northern Africa than in South America	FALSE	184, 49.6%	128, 52.7%	56, 43.8%	0.102

**Table 3:** Univariate analysis of individual factors associated with higher knowledge status (Note: TD = travelers’ diarrhea).

	Knowledge Status on Travelers Diarrhea		*p* Value (Chi-Squared Test)
	High (No./67, %)	Low (No./304, %)	
Individual factors			
Male sex	42, 62.7%	119, 39.1%	<0.001
Age ≥ 35 years	53, 79.1%	212, 69.7%	0.124
Seniority ≥ 10 years	41, 61.2%	165, 54.3%	0.302
Working as General Practitioner	9, 13.4%	41, 13.5%	0.991
Background qualification in Hygiene and Public Health	18, 26.9%	21, 6.9%	<0.001
Professional encounters with TD			
Managed at least one case of TD in the last 12 months	42, 62.7%	156, 51.3%	0.091
Involved in preventive consultations in the previous 12 months	15, 22.4%	83, 27.3%	0.409
Previously affected by TD	20, 29.9%	114, 37.5%	0.238
Recognizing TD as a frequently reported disorder	60, 89.6%	163, 53.6%	<0.001
Recognizing TD as a severe disorder	33, 49.3%	89, 29.3%	0.002
Practices (often to always)			
Recommends antimicrobial prophylaxis	0, -	13, 4.3%	0.085
Recommends antimicrobial treatment	0, -	7, 2.3%	0.210
Questionnaire collected in 2022	24, 35.8%	104, 34.2%	0.802

**Table 4:** Multivariate analysis of factors associated with higher knowledge status. The model included all variables that, in univariate analyses, were associated with a better knowledge score having *p* < 0.05 alongside a subset of factors that were included “a priori” (gender, age, having previously managed TD cases, and having completed the questionnaire in 2022).

	Higher Knowledge Status	
	aOR	95%CI
Male gender	3.509	0.807; 6.814
Age ≥ 35 years	1.058	0.516; 2.169
Background qualification Hygiene and Public Health	14.769	5.582; 39.073
Previously managed TD	3.107	1.484; 6.506
Recognizing TD as a frequently reported disorder	8.579	3.383; 21.756
Recognizing TD as a severe disorder	3.386	1.741; 6.585
Questionnaire collected in 2022	1.916	0.931; 3.941

Note: TD = travelers’ diarrhea; aOR = adjusted Odds Ratio; 95%CI = 95% Confidence Interval.

## Data Availability

Original questionnaire can be freely shared and modified by the end users (See Appendix B Table A2).

## Appendix A

**Table A1:** Strobe statement Checklist.

	Item No	Recommendation	Page No.
**Title and Abstract**			
	1	(*a*) Indicate the study’s design with a commonly used term in the title or the abstract	1
		(*b*) Provide in the abstract an informative and balanced summary of what was done and what was found	1
**Introduction**			
Background/rationale	2	Explain the scientific background and rationale for the investigation being reported	2,3
Objectives	3	State specific objectives, including any prespecified hypotheses	2,3
**Methods**			
Study design	4	Present key elements of study design early in the paper	3
Setting	5	Describe the setting, locations, and relevant dates, including periods of recruitment, exposure, follow-up, and data collection	2–4
Participants	6	(*a*) Give the eligibility criteria, and the sources and methods of selection of participants	2
Variables	7	Clearly define all outcomes, exposures, predictors, potential confounders, and effect modifiers. Give diagnostic criteria, if applicable	2–4
Data sources/measurement	8 *	For each variable of interest, give sources of data and details of methods of assessment (measurement). Describe comparability of assessment methods if there is more than one group	3,4
Bias	9	Describe any efforts to address potential sources of bias	3
Study size	10	Explain how the study size was arrived at	4
Quantitative variables	11	Explain how quantitative variables were handled in the analyses. If applicable, describe which groupings were chosen and why	4
Statistical methods	12	(*a*) Describe all statistical methods, including those used to control for confounding	4
		(*b*) Describe any methods used to examine subgroups and interactions	4
		(*c*) Explain how missing data were addressed	4
		(*d*) If applicable, describe analytical methods taking account of sampling strategy	3
		(*e*) Describe any sensitivity analyses	-
**Results**			
Participants	13 *	(a) Report numbers of individuals at each stage of study—eg numbers potentially eligible, examined for eligibility, confirmed eligible, included in the study, completing follow-up, and analysed	5
		(b) Give reasons for non-participation at each stage	5
		(c) Consider use of a flow diagram	5
Descriptive data	14 *	(a) Give characteristics of study participants (e.g., demographic, clinical, social) and information on exposures and potential confounders	6
		(b) Indicate number of participants with missing data for each variable of interest	5,6
Outcome data	15 *	Report numbers of outcome events or summary measures	7
Main results	16	(*a*) Give unadjusted estimates and, if applicable, confounder-adjusted estimates and their precision (e.g., 95% confidence interval). Make clear which confounders were adjusted for and why they were included	7–10
		(*b*) Report category boundaries when continuous variables were categorized	10
		(*c*) If relevant, consider translating estimates of relative risk into absolute risk for a meaningful time period	-
Other analyses	17	Report other analyses done—e.g., analyses of subgroups and interactions, and sensitivity analyses	Annex 2,3
**Discussion**			
Key results	18	Summarise key results with reference to study objectives	12
Limitations	19	Discuss limitations of the study, taking into account sources of potential bias or imprecision. Discuss both direction and magnitude of any potential bias	14,15
Interpretation	20	Give a cautious overall interpretation of results considering objectives, limitations, multiplicity of analyses, results from similar studies, and other relevant evidence	12–14
Generalisability	21	Discuss the generalisability (external validity) of the study results	14
**Other Information**			
Funding	22	Give the source of funding and the role of the funders for the present study and, if applicable, for the original study on which the present article is based	16

* Give information separately for exposed and unexposed group.

## Appendix B

### Author’s Translation of the Informed Consent

Estimated colleague, the present survey has been developed and shared with the aim of assessing the knowledge of medical professionals on Travelers’ Diarrhea (TD) and its main preventive/treatment options. The present survey specifically targets professionals having a medical and only has scientific aims. In order to thank you for your cooperation, at the end of the questionnaire (whose items are based on the available scientific evidence), the final score will be shown alongside the detailed answers to the reported questions.

While we thank you for your cooperation, we stress that web-based surveys must fulfill the requirements represented by the “Helsinki protocol” and EU Regulation 2016/679.

In order to fulfill the requirements of the Helsinki protocol, we are requesting to formally share your consent. Without your consent, the survey will not continue. Even after your consent, you can leave the present survey at any moment until the sharing of the questionnaire (button “share module” at the end of the questionnaire). Moreover, we stress that the questionnaire will be registered in an anonymous form, and in no way could it be associated with the compiler, as we will not retain any specific, individual information (e.g., email address or IP address of your computer). All requested personal data are generic ones and functional to the demographic analyses (gender, age, etc.).

According to the EU Regulation 2016/279 (GDPR), we also state that:

(1) Data controller, processor, as well as responsible for their retention during the analyses will be Dr. **********, whom you can ask about the process through his personal email (*********). Collected data are generic ones, with SOLE SCIENTIFIC AIMES that have been previously reported. Please be aware that all personal data must be shared with Criminal Law Authorities, without previous personal consent; in the cases that are specifically reported by the current legal framework, without a specific request, retrieved will not be shared with third parties.
(2) After the completion of the questionnaire, we cannot identify in any way the compiler; as the questionnaire is totally anonymous by design, we cannot perform any modification, correction of data collected, and their removal as well.
(3) Data will be retained only for the time strictly required for the aforementioned analyses.

Do you agree to participate in the present survey? YES/NO

(4) Are you a Medical Professional licensed to practice Occupational Medicine in Italy? YES/NO

**Table A2:** Authors’ translation of the items included in the questionnaire.

Section 1. Your personal experience with TD…	
Have you managed any TD case in the previous 12 months?	[YES] [NO] [NO ANSWER]
Have you been involved in pre-travel consultations in the previous 12 months?	[YES] [NO] [NO ANSWER]
Have you been previously affected by TD?	[YES] [NO] [NO ANSWER]
**Section 2. At your knowledge (please mark the correct answer)**	
1. Usually, travelers’ diarrhea resolves spontaneously in 3 to 5 days	[TRUE] [FALSE] [DON’T KNOW]
2. Fluoroquinolones are specifically recommended for patients at high risk of medical complications	[TRUE] [FALSE] [DON’T KNOW]
3. Rifaximin should be preventively employed in patients at high risk of complications	[TRUE] [FALSE] [DON’T KNOW]
4. Fluoroquinolone antibiotics should be avoided as first- line option in patients affected by travelers’ diarrhea	[TRUE] [FALSE] [DON’T KNOW]
5. Rifaximin may be used to treat severe, non-dysenteric travelers’ diarrhea	[TRUE] [FALSE] [DON’T KNOW]
6. There is insufficient evidence to recommend the use of commercially available prebiotics or probiotics to prevent or treat travelers’ diarrhea	[TRUE] [FALSE] [DON’T KNOW]
7. Anti-cholera immunization is somewhat protective for travelers’ diarrhea	[TRUE] [FALSE] [DON’T KNOW]
8. Anti-rotavirus immunization is somewhat protective for travelers’ diarrhea	[TRUE] [FALSE] [DON’T KNOW]
9. Treating liquids/beverages at 100°C for 1′ reduces risk of travelers’ diarrhea	[TRUE] [FALSE] [DON’T KNOW]
10. Consumption of ice drinks reduces risk of travelers’ diarrhea	[TRUE] [FALSE] [DON’T KNOW]
12. Most infections associated with travelers’ diarrhea are of bacterial etiology	[TRUE] [FALSE] [DON’T KNOW]
13. The most frequently reported cause of watery diarrhea is…	[ ] *Campylobacter* spp.
	[ ] *Escherichia coli* (ETEC)
	[ ] Amoebic infections
	[ ] Norwalk virus infections
	[ ] *Shigella* spp.
	[ ] *Vibrio cholerae*
	
14. The most frequently reported cause of dysentery is…	[ ] *Campylobacter* spp.
	[ ] *Escherichia coli* (ETEC)
	[ ] Amoebic infections
	[ ] Norwalk virus infections
	[ ] *Shigella* spp.
	[ ] *Vibrio cholerae*
	
15. The most frequently reported cause of gastrointestinal syndrome characterized by diarrhea and vomiting is…	[ ] *Campylobacter* spp.
	[ ] *Escherichia coli* (ETEC)
	[ ] Amoebic infections
	[ ] Norwalk virus infections
	[ ] *Shigella* spp.
	[ ] *Vibrio cholerae*
	
16. Use of loperamide and analogous is not supported by available evidence	[TRUE] [FALSE] [DON’T KNOW]
17. Bismuth Subsalicylate (BSS) may be considered for any traveler to prevent travelers’ diarrhea	[TRUE] [FALSE] [DON’T KNOW]
18. Fresh fruits/vegetables reduce risk of travelers’ diarrhea	[TRUE] [FALSE] [DON’T KNOW]
19. Drinks on tap are associated with reduced risk of travelers’ diarrhea	[TRUE] [FALSE] [DON’T KNOW]
20. A typhoid vaccine is available in Italy	[TRUE] [FALSE] [DON’T KNOW]
21. Globally, incidence of travelers’ diarrhea is decreasing	[TRUE] [FALSE] [DON’T KNOW]
22. The geographic area at highest risk of travelers’ diarrhea is Southern Asia	[TRUE] [FALSE] [DON’T KNOW]
23. Risk of travelers’ diarrhea is usually higher in Northern Africa than in South America	[TRUE] [FALSE] [DON’T KNOW]
**Section 3. Please rate the following items** **from “not significant” (1) to “very significant” (5)**	
Ho do you perceive the frequency of TD in occupational settings?	[1] [2] [3] [4] [5]
Ho do you perceive the severity of TD in occupational settings?	[1] [2] [3] [4] [5]
**Section 4. In the previous months…** (Notes: 1, “never” = i.e., you did deliberately exclude the use of antimicrobial prophylaxis/treatment in all consultations; 2, “rarely” = antimicrobial prophylaxis/treatment as usually avoided, but rare exceptions are possible; 3, “sometimes” = prophylaxis/treatment is usually avoided, but selected cases may still be targeted; 4, “often” = prophylaxis/treatment is recommended in the majority of travelers, according to their clinical conditions and environmental features of the travel; 5, “always” = prophylaxis/treatment is recommended to all travelers).	
Have you recommended any antimicrobial prophylaxis for overseas workers?	[1] [2] [3] [4] [5]
Have you recommended any antimicrobial treatment for TD in overseas workers?	[1] [2] [3] [4] [5]
**Section 5. Please provide some general information about you**	
Year of birth:	______________
Year of medical qualification:	______________
Are you practicing as General Practitioner alongside Occupational Physician?	[Yes] [No] [No Answer]
Background qualification in Hygiene and Public Health?	[Yes] [No] [No Answer]
You identify yourself as:	[Male] [Female] [No Answer]

Notes: the present questionnaire can be shared and modified by the end user. Please cite the present paper.

**Table A3:** Univariate analysis of individual factors associated with having had any previous experience with TD, either as having been affected by TD of having managed at least one case of TD in the previous 12 months (Note: TD = *travelers’ diarrhea*).

	Any Previous Interaction with TD		*p* Value (Chi-Squared Test)
	YES (No./249, %)	NO (No./122, %)	
Individual factors			
Male sex	106, 42.6%	55, 45.1%	0.647
Age ≥ 35 years	183, 73.5%	82, 67.2%	0.208
Seniority ≥ 10 years	149, 59.8%	57, 46.7%	0.017
Working as General Practitioner	43, 17.3%	7, 5.7%	0.002
Background qualification in Hygiene and Public Health	11, 4.4%	28, 23.0%	<0.001
Participating in pre-travel consultations (in the previous 12 months)	93, 37.3%	5, 4.1%	<0.001
General Knowledge Score > median (65.2%)	42, 16.9%	25, 20.5%	0.394
Recognizing TD as a frequently reported disorder	165, 66.3%	58, 47.5%	0.001
Recognizing TD as a severe disorder	55, 22.1%	67, 54.9%	<0.001
Practices (often to always)			
Recommends antimicrobial prophylaxis	6, 2.4%-	7, 5.7%	0.101
Recommends antimicrobial treatment	0, -	7, 5.7%%	<0.001
Questionnaire collected in 2022	69, 27.%	59, 48.4%	<0.001

**Table A4:** Univariate analysis of individual factors associated with participating in pre-travel consultations (Note: TD = travelers’ diarrhea).

	Participating in Pret-Ravel Consultations (in the Previous 12 Months)		*p* Value (Chi-Squared Test)
	YES (No./98, %)	NO (No./273, %)	
Individual factors			
Male sex	50, 51.0%	111, 40.7%	0.076
Age ≥ 35 years	73, 74.5%	192, 70.3%	0.434
Seniority ≥ 10 years	59, 60.2%	147, 53.8%	0.277
Working as General Practitioner	22, 22.4%	28, 10.3%	0.002
Background qualification in Hygiene and Public Health	0, -	39, 14.3%	<0.001
Professional encounters with TD			
Managed at least one case of TD in the last 12 months	78, 79.6%	120, 44.0%	<0.001
Previously affected by TD	57, 58.2%	77, 28.2%	<0.001
General Knowledge Score > median (65.2%)	15, 15.3%	52, 19.0%	0.409
Recognizing TD as a frequently reported disorder	55, 56.1%	168, 61.5%	0.348
Recognizing TD as a severe disorder	15, 15.3%	107, 39.2%	<0.001
Practices (often to always)			
Recommends antimicrobial prophylaxis	0, -	13, 4.8%	0.028
Recommends antimicrobial treatment	0, -	7, 2.3%	0.110
Questionnaire collected in 2022	20, 20.4%	108, 39.6%	0.001
